# GC-MS, LC-MS, and network pharmacology analysis to investigate the chemical profiles and potential pharmacological activities in flower buds and flowers of *Lonicera japonica* Thunb

**DOI:** 10.1371/journal.pone.0320293

**Published:** 2025-04-23

**Authors:** Kai Tong, Liangli Dai, Wenhui Rui, Yinhao Zhang, Jimei Fu, Yuxue Liao, Wenting Wang, Mengsheng Deng, Yadong Mi, Zhaoling Li

**Affiliations:** 1 School of Biological Engineering, Sichuan University of Science & Engineering, Yibin, China; 2 Bazhong Academy of Agriculture and Forestry Sciences, Bazhong, China; China Three Gorges University, CHINA

## Abstract

*Lonicera japonica* Thunb. (*L. japonica*) is an edible-medicinal herb. While the flower buds of *L. japonica* are commonly utilized for medicinal purposes, the flowers are often overlooked. However, it has been discovered that the flowers contain higher levels of certain active compounds compared to the flower buds. Despite this finding, there have been no reports on the potential differences in pharmacological efficacy between these compounds. Utilizing results from GC-MS and LC-MS, a total of 335 differential compounds were identified, of which 247 complied with Lipinski’s Rule of Five concerning medicinal properties. Among these, 101 compounds were upregulated in the flower buds, while 146 compounds were upregulated in the flowers. Network pharmacology analysis revealed that the upregulated compounds from the flower buds and flowers targeted 143 and 185 core targets, respectively, with 116 being duplicates. The core target proteins among the duplicate targets were primarily involved in pathways related to cancer, lipid and atherosclerosis, hepatitis B, proteoglycans in cancer, and Alzheimer’s disease. Meanwhile, the hub target proteins upregulated in the flowers enriched distinct pathways associated with human T-cell leukemia virus 1 infection, focal adhesion, the thyroid hormone signaling pathway, and fluid shear stress and atherosclerosis. Molecular docking results indicated that the upregulated compounds exhibited strong binding affinity to the core targets. This study provides insights into the differences in active components between the medicinal (flower buds) and non-medicinal (flowers) raw materials predicting the mechanisms of action of these active components and establishing a basis for the more rational utilization of *L. japonica* flowers.

## Introduction

*Lonicera japonica* Thunb. (*L*. japonica), a member of the Caprifoliaceae family, is extensively cultivated in East Asian countries, including China, Japan, and Korea [1]. *L*. japonica is an edible medicinal herb that has been utilized for thousands of years and is commonly known as Japanese honeysuckle, Jin Yin Hua or Ren Dong. Since 1995, *L*. japonica has been included in the Pharmacopoeia of the People’s Republic of China where it is recognized for its antibacterial, anti-inflammatory, antiviral, and other pharmacological properties [2,3]. The flower buds of *L. japonica* are extensively employed in the treatment of various ailments, including hepatitis, throat inflammations, infected wounds, gastroenteritis, SARS coronavirus, and the H1N1 flu virus [4–6]. Furthermore, *L. japonica* flower buds are also utilized as a health-promoting beverage to enhance overall wellness and prevent illnesses, manifesting in products such as “Jin Yin Hua tea”, “Jin Yin Hua nutritional beverage”, and “Jin Yin Hua wine” [7]. These applications illustrate the potential of *L. japonica* as a health food, functional food, and nutritional supplement [8].

The historical emphasis on the medicinal properties of the flower buds has resulted in the oversight and underutilization of the flowers of *L*. *japonica*. Several studies have conducted comprehensive comparisons of the chemical components in various medicinal parts of *L. japonica* using LC-MS analysis [1,9]. The results indicate a rich diversity of chemical components among the different medicinal tissues. Variations in the composition or concentration of these chemical components are the primary reasons for the differences in efficacy of various herbal materials derived from the same medicinal plant. For instance, the flowers of *L. japonica* exhibit higher levels of volatile oils, which demonstrate inhibitory effects on foodborne pathogens such as *Listeria mononuclear* (ATCC 19116), *Bacillus subtilis* (ATCC 6633), *Bacillus cereus* (SCK 11), and *Staphylococcus aureus* (ATCC 6538 and KCTC 1916) [5,10]. Additionally, the flowers contain elevated levels of specific sugars and anthocyanins compared to the flower buds, including mannose, tagatose, galactose, arabinose, cyanidin-3-O-sophoroside, and cyanidin-3-O-sambubioside [11]. The flowers also encompass numerous active compounds, such as acylated flavanol glucosides, phenolic acids, and iridoids [1]. These chemical components serve as the material basis for the pharmacodynamics of Traditional Chinese medicines. The significant differences in composition between the flower buds and flowers of *L*. *japonica* may contribute to variations in pharmacological activities. However, the distinctions in components and pharmacological activities between the flower buds and flowers of *L*. *japonica* remain unclear.

It is essential to consider the pharmacological activity of compounds in the quality control and utilization of traditional herbal medicine [12]. Network pharmacology is an emerging approach that investigates the integrated efficacies of herbs and enhances efficiency in pharmaceutical development [13,14]. This method has been successfully employed in numerous studies to predict active pharmaceutical compounds for major disease resistance [15–18]. The present study combines GC-MS and LC-MS with network pharmacology to examine the differences in potential active compounds and targets between medicinal (flower buds) and non-medicinal (flowers) raw materials. This research aims to provide theoretical guidance for the quality control, product classification, development, and comprehensive utilization of *L*. *japonica* products.

## Materials and methods

### Plant materials

Samples of *L. japonica* flower buds and flowers were collected from Tianquan County, Ya’an City, Sichuan Province, China (E30^º^ 8′3′, N102^º^ 48′26′). The samples were authenticated by Professor Meng-liang Tian, an expert in medicinal botany at the College of Agronomy, Sichuan Agricultural University. Our study site did not involve endangered or protected species; therefore, no specific permissions were required for the location or activity.

### GC-MS analysis

For the GC-MS analysis, 10 mg of the powdered sample was accurately weighed and placed into 2 mL Eppendorf (EP) tubes. The sample was then mixed with 450 μL of a methanol/H2O (3:1, v/v) extraction solution. An internal standard, adonitol (0.5 mg/mL stock in dH_2_O), was added to the tube, and the mixture was vortexed for 30 seconds. The samples were homogenized in a ball mill for 4 minutes at 45 Hz, followed by ultrasound treatment for 5 minutes while incubated in ice water. After centrifugation at 12,000 rpm for 15 minutes at 4 °C, the supernatant was transferred to fresh 1.5 mL EP tubes. The extracts were freeze-dried using a refrigerated centrifugal vacuum concentrator. The samples were then re-dissolved in 80 μL of methoxyamination hydrochloride (20 mg/mL in pyridine), and incubated at 80 °C for 30 minutes. Subsequently, 100 μL of the BSTFA reagent (1% TMCS, v/v) was added to the sample aliquots and incubated at 70 °C for 1.5 hours. Finally, all samples were analyzed using a gas chromatograph system coupled with a Pegasus HT time-of-flight mass spectrometer (GC-MS).

GC-MS analysis was conducted using an Agilent 7890 gas chromatograph system coupled with a Pegasus HT time-of-flight mass spectrometer. The analysis utilized a DB-5MS capillary column, coated with 5% diphenyl and 95% dimethylpolysiloxane (30 m × 250 μm inner diameter, 0.25 μm film thickness; J&W Scientific, Folsom, CA, USA). A 1 μL aliquot of the analyte was injected in splitless mode. Helium served as the carrier gas, with a front inlet purge flow of 3 mL min^−1^ and a gas flow rate of 1 mL min^−1^ through the column. The initial temperature was maintained at 50 °C for 1 minute, followed by an increase to 310 °C at a rate of 10 °C min^−1^, after which it was held at 310 °C for 8 minutes. The temperatures for the injection port, transfer line, and ion source were set at 280 °C, 280 °C, and 250 °C, respectively. Electron impact ionization was employed with an energy of -70 eV. Mass spectrometry data were acquired in full-scan mode, covering the m/z range of 50–500 at a rate of 12.5 spectra per second, following a solvent delay of 6.17 minutes.

The Chroma TOF 4.3X software from LECO Corporation, in conjunction with the LECO-Fiehn Rtx5 database, was utilized for extracting raw peaks, filtering and calibrating data baselines, aligning peaks, performing deconvolution analysis, identifying peaks, and integrating peak areas [19]. Missing values in the raw data were replaced with values equivalent to half of the minimum observed value. Both mass spectrum matching and retention index matching were utilized for metabolite identification. Peaks detected in fewer than 50% of samples, or those with a relative standard deviation (RSD) greater than 30% in quality control (QC) samples, were excluded from analysis [20]. Furthermore, the internal standard normalization method was employed for data analysis [21].

### LC-MS analysis

For the LC-MS analysis, plant samples (20 mg ± 1 mg) were lyophilized and subsequently mixed with beads and 1000 μL of an extraction solution composed of methanol, acetonitrile, and water in a 2:2:1 (v/v) ratio. This extraction solution included deuterated internal standards. The mixture was vortexed for 30 seconds, followed by homogenization at 35 Hz for 4 minutes and sonication for 5 minutes in a 4 °C water bath, with this step repeated three times. The samples were then incubated for 1 hour at -40 °C to precipitate proteins. Subsequently, the samples were centrifuged at 12,000 rpm (RCF = 13800 (×g), R = 8.6 cm) for 15 minutes at 4 °C. A volume of 400 μL of the supernatant was transferred to a protein precipitation plate. The plate was then placed on the manifold, and a vacuum of 6 psi was applied for 120 seconds. Finally, the plate was removed from the positive pressure device for analysis.

The Orbitrap Exploris 120 mass spectrometer was employed for its capability to acquire MS/MS spectra in information-dependent acquisition (IDA) mode, controlled by the acquisition software Xcalibur (Thermo). In this mode, the acquisition software continuously evaluates the full scan MS spectrum. The conditions for the electrospray ionization (ESI) source were set as follows: sheath gas flow rate at 50 Arb, auxiliary gas flow rate at 15 Arb, capillary temperature at 320 °C, sweep gas at 1 Arb, vaporizer temperature at 350 °C, full MS resolution at 60,000, MS/MS resolution at 15,000, and collision energy set to SNCE 20/30/40. The spray voltage was adjusted to 3.8 kV for positive ionization and -3.4 kV for negative ionization.

### Amino acid data determination

The sample preparation for amino acid analysis was conducted as follows: A 100 mg powder sample was transferred into 10 mL EP tubes and mixed with 4 mL of 4% sulfosalicylic acid. The resulting mixture was sonicated at 60 °C for 30 minutes. After allowing it to settle for 10 minutes, 1.5 mL of the supernatant was transferred into a fresh 2.0 mL EP tube. The solution was then centrifuged at 12,000 rpm for 40 minutes and filtered using 0.22 µm membrane filters. Finally, 200 µL of the obtained filtrate was transferred into a brown bottle for further testing.

The amino acid analysis was conducted using an automatic amino acid analyzer system (Hitachi L-8900, Japan) [22]. The analysis utilized a chromatographic column (4.6 × 60 mm) packed with 3 µm sulfonic acid cation exchange resin. The reactor temperature was maintained at 130 °C, while the column temperature was set at 38 °C. A sample injection volume of 20 mm^3^ was employed [22]. The flow rates for the mobile phase and derivatization reagent were 0.35 cm^3^/min and 0.3 cm^3^/min, respectively. Detection was performed at wavelengths of 570 nm and 440 nm for the first and second channels, respectively.

### Multivariate statistical analysis

To compare the chemical compounds in *L. japonica* flower buds and flowers, multivariate statistical analysis was conducted on the sample data. Analysis of variance (ANOVA) was employed to identify compounds that exhibited significant differences between the two groups. Additionally, the SIMCA 18.0.1 software package (Sartorius Stedim Data Analytics AB, Umea, Sweden) was employed to conduct principal component analysis (PCA) and orthogonal partial least squares discriminant analysis (OPLS-DA). Hierarchical clustering analysis (HCA) was conducted using an online tool (https://www.bioinformatics.com.cn/). Compounds with a q-value < 0.05 and a variable importance of projection (VIP) > 1 were deemed significant for sample differentiation [15,23,24].

### Network pharmacological analysis

Potential active compounds were selected from the identified compounds using GC-MS and LC-MS, and their molecular structures were confirmed via PubChem [12]. The Swiss Target Prediction Database (http://www.swisstargetprediction.ch/), STITCH Database (http://stitch.embl.de/), and TCMSP Database (http://ibts.hkbu.edu.hk/LSP/tcmsp.php) were utilized to construct a target database for the potential medicinal compounds [24,25]. Only targets corresponding to “Homo sapiens” were selected [12]. The results were summarized, deduplicated, merged, and transformed into standard gene names in the UniProt database [25]. The protein targets were imported into the online platform STRING (https://string-db.org/) to investigate known and predicted interactions between proteins [26]. The specific screening criteria included selecting “Homo sapiens” in the organism column, choosing “evidence” in the network edges column, and setting the confidence level to > 0.9 [17]. All network results, including the “compound-target” network, “protein-protein interactions (PPI)” network, and “target-pathway” network, were analyzed and visualized using Cytoscape 3.9.1. The degree, betweenness, and closeness of the network were analyzed using the “Network Analyzer” tool [24]. In this context, the nodes and edges represent the targets and their connections, respectively [13]. The obtained target information was imported into the DAVID database for Gene Ontology (GO) and Kyoto Encyclopedia of Genes and Genomes (KEGG) enrichment analysis. The GO enrichment analysis encompassed biological process (BP), cellular component (CC), and molecular function (MF). A significance level of *P-value* < 0.05 was considered in this study.

### Molecular docking of key compound-core target

The SDF format file of the core compound was downloaded from the PubChem database (https://pubchem.ncbi.nlm.nih.gov/), while the Protein Data Bank (PDB) file for the target protein structure was obtained from the PDB database. The target proteins were dehydrated and de-liganded using PyMOL. Autodock Tools software was employed for hydrogenation and charge computation, resulting in the generation of a pdbqt format file. The pdbqt files of both the receptor and ligand were subsequently imported into Autodock Tools for molecular docking. Following this, analysis and visualization were conducted using PyMOL software. The binding affinity was assessed based on the binding energy between the receptor and ligand with a binding energy of < 0 kcal/mol indicating spontaneity, and <-4.25 kcal/mol signifying good binding [27].

### Statistical analyses

Analysis of variance was conducted using SPSS version 21.0 (IBM, USA). Data are presented as means ± standard deviation (SD), with error bar representing the standard deviation. Statistical analyses were performed using a two-tailed Student’s t-test, with a significance level set at *p* ≤ 0.05.

## Results

### Chemical constituent analysis of *L. japonica* flower buds and flowers

**Fig 1:**
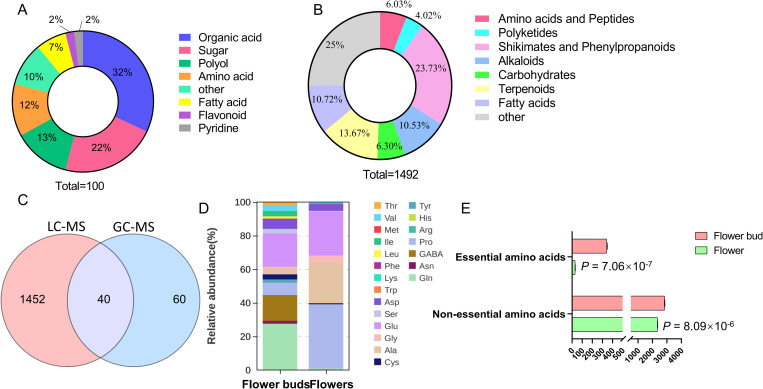
Compound profiles of *L. japonica* flower buds and flowers. A: An overview of annotated compounds identified through GC-MS analysis. B: An overview of annotated compounds identified through LC-MS analysis. C: A Venn diagram illustrating the compounds identified by both GC-MS and LC-MS. D: The profiles of amino acids present in the samples. E. The content of essential and non-essential amino acids.

GC-MS combined with LC-MS was employed to conduct a comprehensive analysis and identification of the chemical compounds present in *L. japonica* flower buds and flowers. In the GC-MS analysis, a total of 667 compound peaks were identified in the samples, with 238 being annotated using the LECO-Fiehn Rtx5 database. Compounds with a similarity score below 600 were filtered out, resulting in 100 retained compounds, which are listed in S1 Table. These compounds were categorized into eight groups: organic acids (32%), sugars (22%), polyols (13%), amino acids (12%), fatty acids (7%), flavonoids (2%), pyridines (2%), and others (10%) (Fig 1A). In the LC-MS analysis, peaks with a relative standard deviation (RSD) greater than 30% were excluded, leading to the detection of a total of 1,492 compounds in both *L. japonica* flower buds and flower samples (S2 Table). These compounds were categorized into eight groups: amino acids and peptides (6.03%), polyketides (4.02%), shikimates and phenylpropanoids (23.73%), alkaloids (10.53%), carbohydrates (6.30%), terpenoids (13.67%), fatty acids (10.72%), and others (25.00%) (Fig 1B). Notably, a total of 40 uniform components were detected using both GC-MS and LC-MS techniques (Fig 1C).

The amino acid content was analyzed in the flower buds and flowers of *L. japonica*. In the flower buds, five essential amino acids were identified: threonine (Thr), valine (Val), isoleucine (Ile), leucine (Leu), and phenylalanine (Phe), resulting in a total essential amino acid content of 340.54 μg/g (Figs 1D and 1E). In contrast, the flowers contained three essential amino acids (Val, methionine (Met) and Ile), with a total essential amino acid content of 30.68 μg/g, which is less than one-tenth of that found in the flower buds (Figs 1D and 1E). The total content of nonessential amino acids was 2836.76 μg/g in the flower buds and 2347.91 μg/g in the flowers, comprising 12 and 9 nonessential amino acids, respectively. Notably, the levels of proline (Pro) and alanine (Ala) in the flowers were significantly higher than those in the flower buds, corroborating the results obtained from GC-MS analysis.

### Multivariate statistical analysis

**Fig 2:**
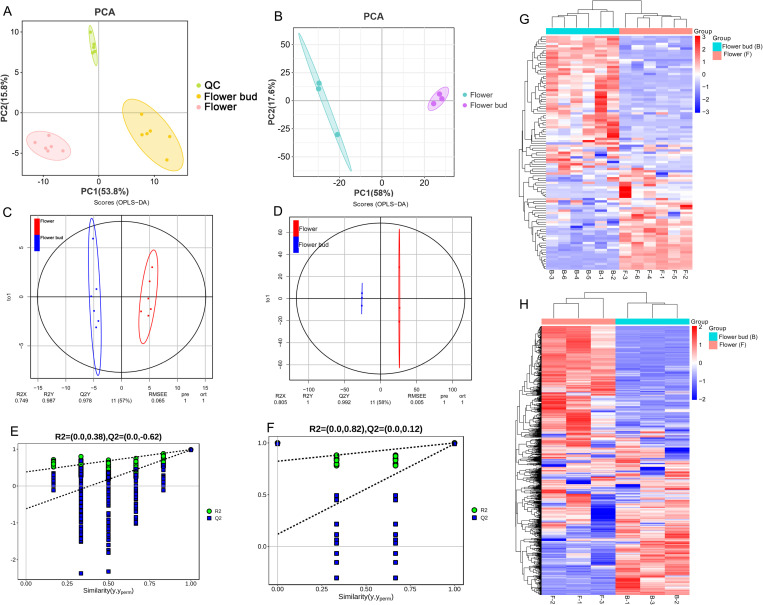
The plot of PCA, OPLS-DA model, and heatmap analyses of *L. japonica* flower buds and flowers. A: The PCA plot of the compound profiles obtained via GC-MS. B: The PCA plot of the compound profiles derived from LC-MS. C: The OPLS-DA score plot based on GC-MS data. D: The OPLS-DA score plot from LC-MS data. E: The corresponding validation plots resulting from 200 times permutation tests of the OPLS-DA model for GC-MS. F: The corresponding validation plots resulting from 200 times permutation tests of the OPLS-DA model for LC-MS. G: The heatmap generated from hierarchical cluster analysis of the compound profiles using GC-MS. H: The heatmap generated from hierarchical cluster analysis of the compound profiles using LC-MS. Columns and rows represent different samples and individual compounds, respectively.

To comprehensively investigate the compound profiling of *L. japonica* flower buds and flowers, PCA and HCA analyses were performed using 100 and 1492 compounds, respectively. The PCA score plots revealed distinct separation trend between *L. japonica* flower buds and flowers. For the GC-MS results, the first and second principal components accounted for 53.8% and 15.8% of the variance, respectively (Fig 2A). Meanwhile, the first and second principal components for LC-MS results explained 58% and 17.6% of the variance, respectively (Fig 2B). Additionally, a heatmap generated from the HCA analysis was utilized to visualize the overall differences between *L. japonica* flower buds and flowers, clearly demonstrating significant disparities between the two groups (Figs 2G and 2H). To further explore the distinct chemical compounds, present in *L. japonica* flower buds and flowers, the OPLS-DA model was utilized. As shown in Figs 2C and 2D, the samples of *L. japonica* flower buds and flowers were distinctly separated on either side of the coordinate axis, highlighting a significant difference in their chemical profiles. The R^2^Y values of the OPLS-DA models for GC-MS and LC-MS were 0.987 and 1, respectively, while the Q^2^ values were 0.978 and 0.992, respectively, demonstrating the strong predictive capacity of the OPLS-DA model (Figs 2C and 2D). Furthermore, 200 permutation tests were conducted to validate the model, with the intercepts of R^2^ and Q^2^ being lower than the original values, indicating a high level of reliability (Figs 2E and 2F).

### Screening of differential compounds in flower buds and flowers of *L. japonica*

**Fig 3:**
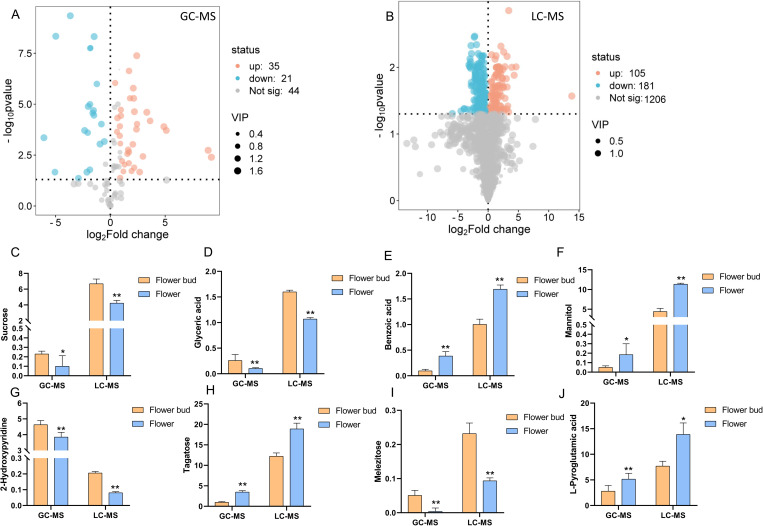
The differential compounds between flower buds and flowers of *L. japonica.* A: The volcano map of the compounds identified by GC-MS. B: Volcano map of the compounds identified by LC-MS. C-J: The duplicated differential compounds identified by both GC-MS and LC-MS. Significant differences in the bar charts are indicated by the number of asterisks: one asterisk (*) denotes **p** < 0.05, while two asterisks (**) denote **p** < 0.01, as determined by Student’s *t-test*.

Based on the criteria of VIP values greater than 1 and q values less than 0.05, a total of 56 and 287 differential compounds were identified by GC-MS and LC-MS analyses, respectively (Figs 3A and 3B). Eight compounds were identified as duplicates, including sucrose, tagatose, mannitol, 2-hydroxypyridine, melezitose, glyceric acid, L-pyroglutamic acid, and benzoic acid, all exhibiting the same trend in content differences between GC-MS and LC-MS (Figs 3C-J). Among these compounds, 136 were upregulated while 198 were downregulated in *L. japonica* flower buds compared to the flowers.

### Prediction of potential active compounds based on network pharmacology

**Fig 4:**
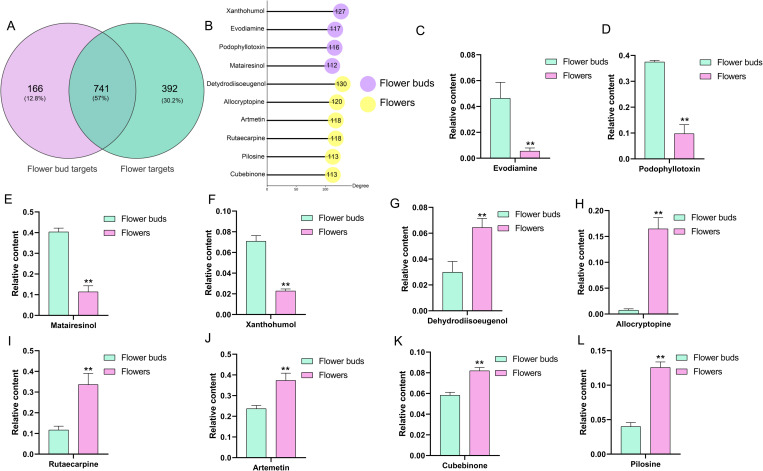
The analysis of the potential targets for the up-regulated compounds found in *L. japonica* flower buds and flowers. A: A Venn diagram illustrates the potential targets identified. B: The top ten compounds exhibiting the highest degrees. C-L: A significance analysis of the content of these top ten compounds. Significant differences in the line charts are indicated by the number of asterisks: one asterisk (*) denotes **p** < 0.05, while two asterisks (**) denote **p** < 0.01, as determined by Student’s *t-test*.

Combining the results from GC-MS and LC-MS, a total of 334 differential compounds were identified, of which 247 met the Lipinski’s Rule of Five concerning medicinal properties (S3 Table). Among these, 101 compounds were up-regulated in flower buds, while 146 compounds were up-regulated in flowers. The varying concentrations of these compounds contributed to differences in the efficacy of *L. japonica* flower buds and flowers. Active compound effect targets were screened using the Swiss Target Prediction Database, STITCH Database, and TCMSP Databases [24]. The resulting network was visualized using Cytoscape 3.7.1 [24]. As shown in S1 Fig, the network comprised a total of 1,547 nodes (including 1300 targets and 247 active components) and 9,262 edges, with 907 and 1,133 potential targets for the up-regulated compounds in *L. japonica* flower buds and flowers, respectively, sharing 741 common targets (Fig 4A). The degree of a node, which indicates its importance within the network, was determined by the number of edges connected to it [28]. The top ten compounds with a degree value ≥ 110 of connection to target proteins were identified as key active compounds, including dehydrodiisoeugenol (Degree=130), xanthohumol (Degree=127), allocryptopine (Degree=120), rutaecarpine (Degree=118), artemetin (Degree=118), evodiamine (Degree=117), podophyllotoxin (Degree=116), cubebinone (Degree=113), pilosine (Degree=113), and matairesinol (Degree=112) (Fig 4B). Notably, among the top ten compounds, six were up-regulated in *L. japonica* flowers, indicating that the up-regulated compounds in *L*. *japonica* flowers interacted with a greater number of targets than those in flower buds (Figs 4C-L).

### PPI network analysis

**Fig 5:**
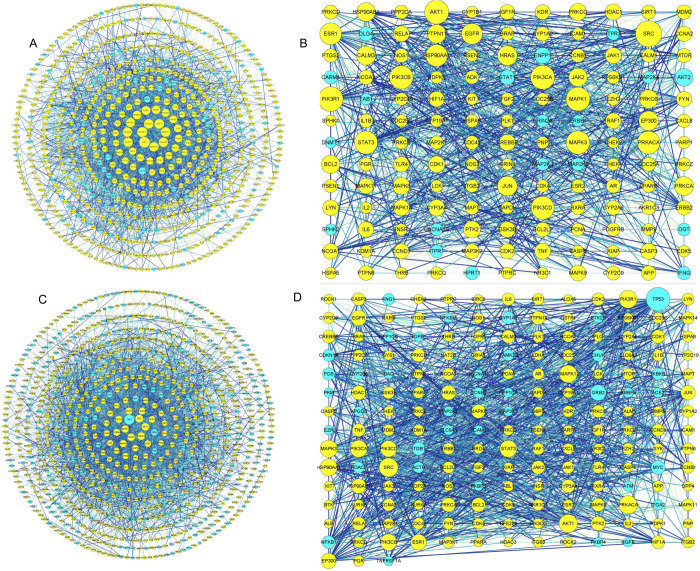
Network maps illustrating the targets of the up-regulated compounds in *L. japonica* flower buds and flowers. A: Network maps of the targets of the up-regulated compounds in *L. japonica* flower buds. B: Network maps of the core targets of the up-regulated compounds in *L. japonica* flower buds. C: Network maps of the targets of the up-regulated compounds in *L. japonica* flowers. D: Network maps of the core targets of the up-regulated compounds in *L. japonica* flowers. The blue nodes represent the unique core targets of the up-regulated compounds in both *L. japonica* flower buds and flowers, while the yellow nodes indicate the shared targets.

To identify core targets, PPI analysis was conducted by submitting the potential targets of active compounds to the STRING database, selecting high-confidence protein interaction data with a score greater than 0.9 [29]. The PPI analysis of the up-regulated compounds in flower buds yielded 708 nodes and 2,722 edges, while the analysis of the up-regulated compounds in flowers resulted in 892 nodes and 3,564 edges (Figs 5A and 5C). The medians of the degree and betweenness centrality values were employed as thresholds to screen for core targets. Based on the degree value, a total of 328 targets were identified as core targets, with 116 being duplicates. Among these, 143 core targets corresponded to the up-regulated compounds in *L. japonica* flower buds, with 27 identified as unique targets (Fig 5B). The top ten core targetsincluded SRC tyrosine protein kinase (SRC, Degree = 57), PI3-kinase p85-alpha subunit (PIK3R1 Degree = 52), serine/threonine protein kinase (AKT1, Degree = 50), PI3-kinase p110-α subunit (PIK3CA, Degree = 50), mitogen-activated protein kinase 1 (MAPK1, Degree = 49), MAP kinase ERK1 (MAPK3, Degree = 48), PI3-kinase p110-β subunit (PIK3CB, Degree = 48), PI3-kinase p110-delta subunit (PIK3 CD, Degree = 47), signal transducer and activator of transcription 3 (STAT3, Degree = 47), and cAMP-dependent protein kinase alpha-catalytic subunit (PRKACA, Degree = 46), which were regarded as core proteins in flower buds (Fig 5B). Similarly, a total of 185 core targets were identified for the up-regulated compounds in *L. japonica* flowers, with 69 being unique targets (Fig 5D). Among these, the top ten targets included cellular tumor antigen p53 (TP53, Degree = 90), SRC (Degree = 63), AKT1 (Degree = 60), PRKACA (Degree = 60), PIK3R1 (Degree = 58), MAPK1 (Degree = 56), MAPK3 (Degree = 54), heat shock protein HSP 90-alpha (HSP90AA1, Degree = 53), PIK3CA (Degree = 53), and STAT3 (Degree = 52) (Fig 5D).

### GO and KEGG enrichment analysis

**Fig 6:**
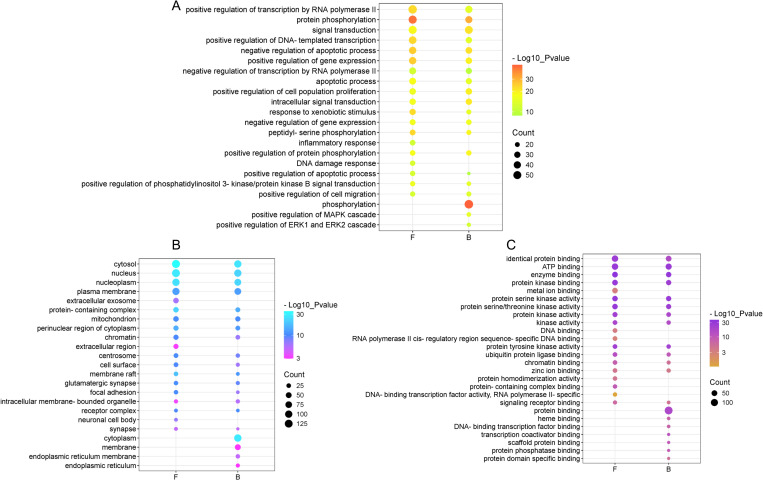
Compare of *L. japonica* flower buds and flowers in GO term associated with *L. japonica* flower buds and flowers. A refers to biological processes. B pertains to cellular component. C relates to molecular functions. B, flower buds; F, flowers.

GO analysis and KEGG data were conducted using the DAVID database for enrichment analysis, with a screening threshold established at *P* < 0.05. A total of 424 GO terms were identified from the up-regulated compounds in *L. japonica* flower buds, comprising 304 biological processes (BP), 43 cellular components (CC), and 77 molecular functions (MF). In contrast, 667 GO terms were identified from the up-regulated compounds in *L. japonica* flowers, including 408 for BP, 52 for CC, and 217 for MF. The top 20 significantly enriched terms across the BP, CC, and MF categories were selected for comparative analysis (Fig 6).

In the BP category, the target proteins of the up-regulated compounds in *L. japonica* flower buds were specifically involved in phosphorylation, the positive regulation of the MAPK cascade, and the positive regulation of the ERK1 and ERK2 cascades (Fig 6A). The up-regulated compounds in the flowers were particularly associated with the inflammatory response and DNA damage response (Fig 6A). In the CC category, the target proteins of the up-regulated compounds in *L. japonica* flower buds were classified as part of the cytoplasm, membrane, endoplasmic reticulum membrane, and endoplasmic reticulum, while those in the flowers were categorized as part of the extracellular exosome, extracellular region, and neuronal cell body (Fig 6B). In the MF category, the target proteins of the up-regulated compounds in *L. japonica* flower buds were specifically involved in protein binding, heme binding, DNA-binding transcription factor binding, transcription coactivator binding, scaffold protein binding, protein phosphatase binding, and protein domain-specific binding (Fig 6C). In contrast, those in the flowers were particularly associated with DNA binding, RNA polymerase II cis-regulatory region sequence-specific DNA binding, protein homodimerization activity, protein-containing complex binding, DNA-binding transcription factor activity, and RNA polymerase II specificity (Fig 6C).

**Fig 7:**
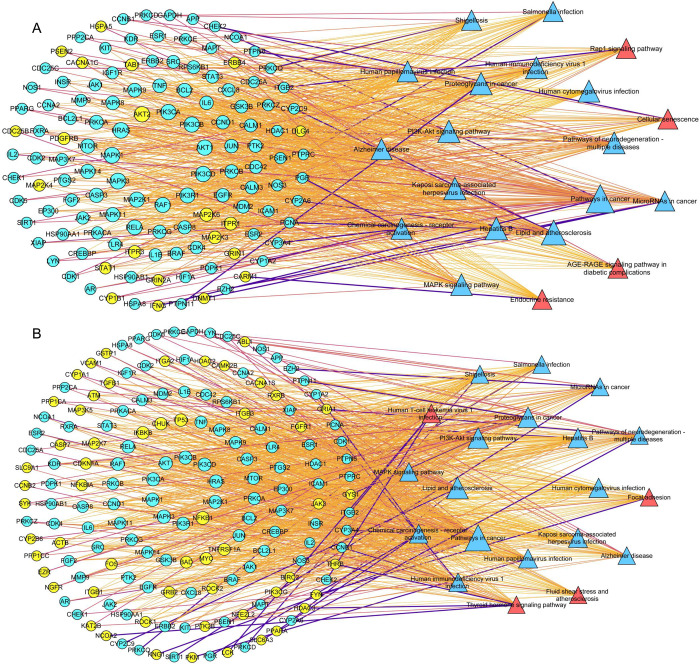
“Target-pathway” network diagram combination of *L. japonica* flower buds and flowers. A: “Target-pathway” network diagram for the up-regulated compounds in *L. japonica* flower buds. B: “Target-pathway” network diagram for the up-regulate compounds in *L*. *japonica* flowers. Yellow and red nodes indicated the unique targets/pathways associated with the up-regulated compounds in *L. japonica* flower buds and flowers, while blue nodes represent shared targets/pathways.

A total of 171 and 174 KEGG pathways were identified for the up-regulated compounds in *L. japonica* flower buds and flowers, respectively. The top 20 pathways selected based on *p*-value, exhibited the highest number of targets. To visualize these findings, we constructed two “target protein-pathway” combination networks for the up-regulated compounds. In these networks, circular nodes represent targets, while triangular nodes denote KEGG pathways. As shown in Fig 7, there were 16 pathways common to the target proteins of up-regulated compounds in both *L. japonica* flower buds and flowers. These pathways included cancer-related pathways, lipid and atherosclerosis pathways, the PI3K-Akt signaling pathway, proteoglycans in cancer, hepatitis B, human cytomegalovirus infection, Kaposi sarcoma-associated herpesvirus infection, human papillomavirus infection, human immunodeficiency virus 1 infection, chemical carcinogenesis-receptor activation, the MAPK signaling pathway, salmonella infection, shigellosis, microRNAs in cancer, Alzheimer’s disease, and pathways related to neurodegeneration-multiple diseases. The target proteins corresponding to the up-regulated compounds in *L. japonica* flower buds were particularly associated with various processes, including the AGE-RAGE signaling pathway in diabetic complication, cellular senescence, the Rap1 signaling pathway, and endocrine resistance (Fig 7A). In contrast, the target proteins associated with the up-regulated compounds in *L. japonica* flowers were specifically enriched in pathways related to human T-cell leukemia virus 1 infection, focal adhesion, the thyroid hormone signaling pathway, and fluid shear stress and atherosclerosis (Fig 7B).

### Molecular docking validation of compound and core targets

**Fig 8:**
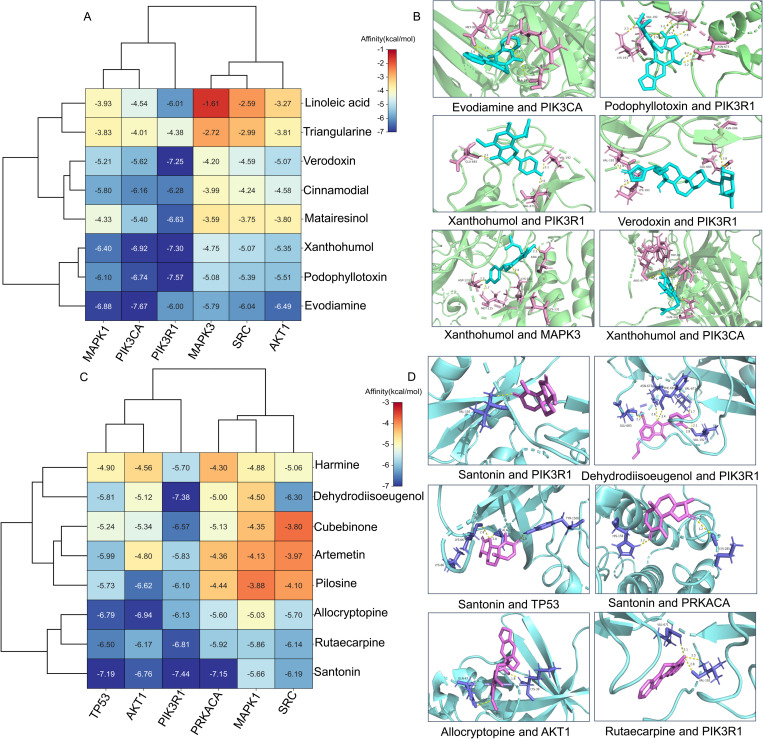
Molecular docking of core compounds and targets. A: The heatmap of the binding energy of core compounds with the target proteins derived from *L. japonica* flower buds. B: Molecular docking models showcasing the interactions between core compounds and target proteins of *L. japonica* flower buds. C: The heatmap depicting the binding energy of core compounds in relation to target proteins of *L. japonica* flower. D: Molecular docking models representing the interactions between core compounds and target proteins of *L. japonica* flower.

The effect of molecular docking is determined by the binding energy between the compound and core targets. In this study, molecular docking was conducted using the AutoDock Tool to evaluate the binding energies between eight active compounds and six core proteins in *L. japonica* flower buds and flowers. The results indicated that the binding energies between the compounds and target proteins were less than 0 kcal/mol, suggesting that the small molecule ligand binds spontaneously to the target protein [27]. A lower binding energy signifies a stronger binding affinity [30]. When the binding energy is less than -4.25 kcal/mol, it indicates relatively favorable binding properties between the receptor and ligand [31]. In this study, the binding energy of all “ligand and receptor” pairs were less than 0 kcal/mol, with 33 and 43 pairs exhibiting binding energies less than -4.25 kcal/mol in flower buds and flowers, respectively, demonstrating strong binding activities (Figs 8A and 8C). In the flower buds of *L. japonica*, the binding energies of four “core compound and core target protein” pairs were less than -7.0 kcal/mol, including evodiamine and PIK3CA, podophyllotoxin and PIK3R1, xanthohumol and PIK3R1, and verodoxin and PIK3R1 (Fig 8A).The molecular docking modes were shown in Fig 8B. Evodiamine formed four hydrogen bonds with ARG-97, ALA-15, and MET-99 residues of PIK3CA. Podophyllotoxin established seven hydrogen bonds with LYS-193, VAL-192, VAL-671, and ASN-673 residues of PIK3R1. Xanthohumol formed three hydrogen bonds with GLU-683, VAL-192, and VAL-671 residues of PIK3R1, and also formed five hydrogen bonds with ASP-123, MET-125, LYS-131, and ASN-171 residues of MAPK3. Additionally, xanthohumol formed six hydrogen bonds with ARG-97, TRP-95, SER-11, ALA-15, and GLN-242 residues of PIK3CA. Verodoxin formed four hydrogen bonds with VAL-192, LYS-193, GLU-683, and ASN-686 residues of PIK3R1 (Fig 8B). In the flowers of *L. japonica*, four “ligand-receptor” pairs exhibited binding energies of less than -7.0 kcal/mol (Fig 8C). These pairs included santonin and PIK3R1, dehydrodiisoeugenol and PIK3R1, santonin and TP53, and santonin and PRKACA. As shown in Fig 8D, santonin formed one hydrogen bond with VAL-19 residue of PIK3R1. Dehydrodiisoeugenol established six hydrogen bonds with GLU-683, ASN-673, PHE-681, VAL-671, and VAL-192 residues of PIK3R1. Santonin also formed three hydrogen bonds with LYS-66, ALA-1555, and TYR-1500 residues of TP53, as well as two hydrogen bonds with HIS-158 and LYS-285 residues of PRKACA. Allocryptopine formed two hydrogen bonds with GLN-47 and LYS-39 residues of AKT1, while rutaecarpine formed three hydrogen bonds with VAL-192 and VAL-671 residues of PIK3R1.These results indicate that the predicted core targets and corresponding active compounds of *L. japonica* flower buds and flowers exhibit strong binding abilities, thereby confirming the reliability of the network pharmacology findings.

## Discussion

In this study, significant similarities were found in the major compounds of the flower buds and flowers of *L. japonica*; however, the content of these compounds varied considerably. These differences in compound content may contribute to the distinct pharmacological effects [32]. Specifically, 247 compounds were identified as significantly differing between the flower buds and flowers of *L. japonica*. Nonetheless, the pharmacological mechanisms underlying these differential compounds remain not fully understood. Consequently, network pharmacology was employed to comprehensively compare the active compounds, action targets, and key pathways. The top ten compounds with a degree value ≥ 110 were identified as key active compounds. Four components were found to be upregulated in flower buds compared to the flowers of *L. japonica*: evodiamine, podophyllotoxin, matairesinol, and xanthohumol. These compounds primarily interact with hub proteins involved in pathways related to cancer, lipid and atherosclerosis, and human immunodeficiency virus 1 infection. Notably, evodiamine, a naturally occurring alkaloid, exhibits numerous pharmacological effects, including anti-inflammatory, anti-cancer, anti-microbial, regulation of metabolic diseases, and anti-neurodegenerative activities [33]. Podophyllotoxin, a naturally occurring lignan, is regarded as an important anticancer compound demonstrating additional immunosuppressive, antiviral, antioxidant, hypolipemic, and anti-inflammatory effects [34]. Xanthohumol, a natural chalcone, has been found to possess various biological effects, including anti-microbial, anti-viral, immunomodulatory, and anti-tumor activities [35].

Meanwhile, the six potential key active compounds identified in the flowers include dehydrodiisoeugenol, allocryptopine, rutaecarpine, artemetin, cubebinone, and pilosine. Dehydrodiisoeugenol, an isoeugenol dimer, exhibits significant hepato-protective, antithrombotic, anti-inflammatory, anti-allergic, anti-oxidant, anti-cancerogenic, and anti-microbial properties [36,37]. Allocryptopine, an isoquinoline alkaloid, has been reported to possess anti-inflammatory and neuroprotective effects [38]. Rutaecarpine, a natural alkaloid, demonstrates a broad spectrum of pharmacological effects, including anti-inflammatory, anti-atherogenic, anti-Alzheimer’s disease, antitumor, and antifungal activities [39,40]. Consequently, the compounds that are highly accumulated in the flowers also exhibit multiple pharmacological activities. Overall, the high accumulation of compounds in both the flower buds and flowers of *L. japonica* results in diverse pharmacological activities due to their interaction with multiple hub target proteins. Therefore, it is crucial to carefully differentiate between flower buds and flowers during the harvesting and processing stages to maintain the stability and efficacy of the quality and therapeutic properties of Chinese medicinal materials. Furthermore, *L. japonica* flowers contain specific active compounds that hold potential for development. Additionally, the flowers are rich in monosaccharides, making them suitable for the development of functional foods, such as beverages, substitute tea, confections, pastries, and other everyday products.

Furthermore, the top 20 pathways exhibited the highest number of targets, with 16 identified as duplicates. For instance, the core target proteins associated with the upregulation of flower buds and flowers of *L. japonica* were enriched in cancer-related pathway, showing counts of 65 and 87, respectively. Consequently, a majority of the hub target proteins of the active compounds in flower buds and flowers were involved in the same pathways. However, four distinct pathways were identified between the hub target proteins of the upregulated compounds in the flower buds and flowers of *L. japonica*. The upregulated compounds corresponding to target proteins in the flower buds of *L. japonica*, including caffeic acid, quinic acid, protocatechuic acid, linoleic acid, and procyanidin B2, were particularly associated with the AGE-RAGE signaling pathway in diabetic complication, cellular senescence, the Rap1 signaling pathway, and endocrine resistance. Notably, caffeic acid, a polyphenol derived from hydroxycinnamic acid, possesses numerous physiological properties, including antioxidant, anti-inflammatory, anti-atherosclerotic, immune-stimulatory, cardioprotective, antiproliferative, and hepatoprotective activities [41]. Caffeic acid also exerts anti-diabetic effects by modulating inflammatory cytokines and transcription factors [41]. Protocatechuic acid has been shown to induce cell death in hepatocellular carcinoma cells via the c-Jun N-terminal kinase pathway [42]. Linoleic acid has been associated with benefits in glycemic control, cardiovascular risk, and insulin resistance [43]. Procyanidin B2, a natural polyphenol, exhibited protective effects against diabetic vasculopathy [44].

The target proteins associated with the up-regulated compounds in *L. japonica* flowers were specifically enriched in pathways related to human T-cell leukemia virus 1 infection, focal adhesion, the thyroid hormone signaling pathway, and fluid shear stress and atherosclerosis. The core proteins, including PIK3CA, PIK3CB, PIK3 CD, PIK3R1, ITGB2, and AKT1, were involved in all of these enriched pathways. Furthermore, the up-regulated compounds in the flowers included phyllanthin and erianin, which interact with multiple hub target proteins associated with human T-cell leukemia virus 1 infection. Phyllanthin, a lignan compound, regulated MOLT-4 cells through the PI-3K/AKT/JNK/MAPK pathway, leading to the inhibition of leukemic cancer [45]. Erianin inhibited the transcriptional level of PIK3R1 by enhancing the protein level of PPAR, thereby suppressing the PI3K/AKT pathway, which resulted in the suppression of acute myeloid leukemia [46].

## Conclusion

In the present study, the flower buds and flowers of *L. japonica* demonstrated significant similarities in their primary compounds; however, the content of these compounds varied considerably. The upregulated compounds in both the flower buds and flowers were primarily associated with a greater number of characteristic targets within the cancer pathway. Furthermore, the targets of the upregulated compounds in the flowers were specifically linked to human T-cell leukemia virus 1 infection, focal adhesion, the thyroid hormone signaling pathway, and fluid shear stress and atherosclerosis. Nonetheless, additional evidence is required to substantiate this hypothesis. Overall, this study provides a comprehensive overview of the differences between the flower buds and flowers of *L. japonica*, serving as a valuable reference for future research on the effective components and pharmacological effects of *L. japonica*.

## Supporting information

S1 Table Detailed information of identified compounds by GC-MS. (XLSX)

S2 Table Detailed information of identified compounds by LC-MS. (XLSX)

S3 Table The active compounds in flower buds and flowers of *L. japonica.* (XLSX)

S1 Fig The “compounds-targets” interaction network model. (TIF)
